# pH Stress-Induced Cooperation between *Rhodococcus ruber* YYL and *Bacillus cereus* MLY1 in Biodegradation of Tetrahydrofuran

**DOI:** 10.3389/fmicb.2017.02297

**Published:** 2017-11-21

**Authors:** Zubi Liu, Zhixing He, Hui Huang, Xuebin Ran, Adebanjo O. Oluwafunmilayo, Zhenmei Lu

**Affiliations:** ^1^College of Life Sciences, Zhejiang University, Hangzhou, China; ^2^College of Basic Medical Science, Zhejiang Chinese Medical University, Hangzhou, China

**Keywords:** metatranscriptome, *Rhodococcus ruber* YYL, *Bacillus cereus* MLY1, cooperation, tetrahydrofuran degradation, low pH stress

## Abstract

Microbial consortia consisting of cooperational strains exhibit biodegradation performance superior to that of single microbial strains and improved remediation efficiency by relieving the environmental stress. Tetrahydrofuran (THF), a universal solvent widely used in chemical and pharmaceutical synthesis, significantly affects the environment. As a refractory pollutant, THF can be degraded by some microbial strains under suitable conditions. There are often a variety of stresses, especially pH stress, that inhibit the THF-degradation efficiency of microbial consortia. Therefore, it is necessary to study the molecular mechanisms of microbial cooperational degradation of THF. In this study, under conditions of low pH (initial pH = 7.0) stress, a synergistic promotion of the THF degradation capability of the strain *Rhodococcus ruber* YYL was found in the presence of a non-THF degrading strain *Bacillus cereus* MLY1. Metatranscriptome analysis revealed that the low pH stress induced the strain YYL to up-regulate the genes involved in anti-oxidation, mutation, steroid and bile acid metabolism, and translation, while simultaneously down-regulating the genes involved in ATP production. In the co-culture system, strain MLY1 provides fatty acids, ATP, and amino acids for strain YYL in response to low pH stress during THF degradation. In return, YYL shares the metabolic intermediates of THF with MLY1 as carbon sources. This study provides the preliminary mechanism to understand how microbial consortia improve the degradation efficiency of refractory furan pollutants under environmental stress conditions.

## Introduction

Bioremediation is an eco-friendly waste management technique that uses naturally-occurring organisms to break down hazardous substances into less toxic or non-toxic substances. Microbial bioremediation has been the most significant remediation method for the mineralization of pollutants in contaminated environments (Vilchez-Vargas et al., 2010; Krastanov et al., 2013). For this reason, many degrading microorganisms have been isolated from the polluted environments and tested for their degradation potential and mechanisms. However, degrading microorganisms do not often exist in mono-culture in the environment. Instead, many bacteria are found in close association with one another and exhibit a superior biodegradation performance than that by a single species (Mikeskova et al., 2012). For example, compared to single bacteria, a consortium of *Pseudomonas* sp. SUK1 and *Aspergillus ochraceus* NCIM-1146 shows an increased ability to break down the textile dye reactive navy blue HE2R in 24 h (Kadam et al., 2011). The biochemical cooperation of different microorganisms accelerates the complete degradation of some pollutants (Wu et al., 2010). In addition, some bacteria may display no degradation ability but protect the bacteria with degradation ability or change the structure of the contaminants to enhance the degradation process (Di Gioia et al., 2004; Zhao and Wong, 2009). Therefore, interactions between microbes play significant roles in hazardous substance degradation.

Tetrahydrofuran (THF) is a heterocyclic ether with the formula (CH_2_)_4_O. As a polar, versatile solvent, THF is widely used as a reaction media for the synthesis of polymers. Additionally, THF is easily detected in the groundwater (Isaacson et al., 2006) and can penetrate the skin, cause rapid dehydration, inhibit cytochrome P450, and induce central nervous system irritation, narcosis, edema, and colonic muscle spasms in animals (Chhabra et al., 1990; Malley et al., 2001). As an eco-friendly and cost-effective strategy for pollutant removal, the application of THF-degrading microorganisms has been rarely investigated. To date, only a few strains of the genera *Rhodococcus* (Daye et al., 2003; Yao et al., 2009; Tajima et al., 2012), *Pseudonocardia* (Kohlweyer et al., 2000; Masuda et al., 2012), and *Pseudomonas* (Chen et al., 2010) have been reported to bear the ability to use THF as the sole carbon source. In our previous studies, we isolated a THF-degrading strain, named *Rhodococcus ruber* YYL, which had a maximum THF degradation rate of 137.60 mg⋅h^-1^⋅g^-1^ YYL dry weight (Yao et al., 2009, 2013). In addition, the symbiotic non-THF-degrading *Bacillus cereus* MLY1 was isolated with strain YYL from the activated sludge and could successfully augment the strain YYL colonization in activated sludge and remarkably improve the THF removal in the reactor (Yao et al., 2013). However, the mechanism of the non-THF-degrading microorganism’s survival in the mineral medium and the interactions with strain YYL remain unclear.

The environmental pH has been one of the key factors affecting the microbial degradation of pollutants (Lowe et al., 1993; Hall-Stoodley et al., 2004; Huertas et al., 2010). Strain YYL shows its optimal degradation efficiency at initial pH 8.3 (Yao et al., 2009). However, actual environment conditions cannot meet this optimal pH. Strain YYL produces acidic substances continuously during the degradation of THF and decreases the pH of its environment, negatively affecting its degradation efficiency. We found that the THF degradation efficiency of YYL could be improved when under low pH stress (initial pH = 7.0) by the addition of the strain MLY1.

To study the interactions between strains YYL and MLY1, these two strains were co-cultured under the initial pH of 7.0 or 8.3 and subjected to metatranscriptome analysis. Here we focus on the difference in THF removal and gene expression in strain YYL and MLY1 between the co-culture and mono-culture systems with different pH conditions.

## Materials and Methods

### Strains, Culture Conditions, and Co-culture Experiments

The THF-degrading strain *R. ruber* YYL was cultured and maintained in 100 mL liquid optimal base mineral medium (BMM) at initial pH of 8.3 with 20 mM THF (Yao et al., 2009); meanwhile, *B. cereus* MLY1 was also cultured and maintained in 100 mL BMM with 1.0 g/L yeast extract. For the initial phase of each co-culture experiment, the stationary phase cells of strain YYL or MLY1 were centrifuged (5 min, 8000 rpm), the growth media was decanted, and the cells were suspended in BMM at the initial pH of 8.3 or 7.0. Subsequently, 100 mL BMM (initial pH = 8.3 or 7.0) with 20 mM THF was inoculated with 2 mL of strain YYL (OD_600_ = 1.5) and 1 mL of MLY1 (OD_600_ = 1.5) to make a cell ratio of 2:1. The corresponding mono-culture systems were incubated with the same inoculum of YYL and 1 mL of BMM. One Erlenmeyer flask of culture was used for each sample, and four replicates were prepared for every sampling time point in the four treatment groups. All the flasks were cultured at 140 rpm and a temperature of 30°C.

### Sample Collection and Detection

Samples were collected for the determination of the THF concentration and the pH during the incubation. THF concentrations were measured by GC-2014C gas chromatography equipped with a flame ionization detector (FID) and an AOC-20i auto injector (SHIMADZU, Japan). The injector, oven, and detector temperatures were set to 200, 160, and 200°C, respectively. The THF peak was observed at a retention time of 1.7 min. For the transcriptional analysis, the sampling time for the experiment was chosen based on the THF removal efficiency and strain growth stage in the four treatment groups. In this study, samples were collected at the initial stage, the first 4 days, during which the cells of all the treatments were at the exponential growth phase. Also, the transcriptional changes in strain YYL that could describe its response within the symbiotic system were identified. All the collected samples were immediately centrifuged at 4°C, flash-frozen in liquid nitrogen, and stored at -80°C. To detect strain MLY1 growth, the ratio of the DNA levels of the specific genes *thm/GerM* were quantified using quantitative PCR (qPCR) to detect the ratio of strain YYL to strain MLY1 in co-culture (Rosenthal et al., 2011; Benomar et al., 2015); *thm* is the THF monooxygenase gene responsible for the first step of THF degradation as described in previous studies (He et al., 2014), and the *GerM* gene encodes a lipoprotein that stabilizes the GerA-GerQ complex through an interaction with the remodeled cell wall during spore formation in bacilli (Rodrigues et al., 2016). The primers for the qPCR are shown in Supplementary Table S1.

### RNA Isolations, Library Construction, and Sequencing

Total RNA was extracted from four biological replicates per growth condition using the RNeasy Mini Kit (Cat#74106, QIAGEN, Germany) according to the manufacturer’s instructions. The RNA quality and yield were measured with gel electrophoresis using 1% agarose gel and NanoDrop 2000 Spectrophotometer (Thermo Scientific, Wilmington, DE, United States). Subsequently, the extracted quadripartite RNA was pooled equally and purified with the RNeasy Micro Kit (Cat#74004, QIAGEN, Germany) and the RNase-Free DNase Set (Cat#79254, QIAGEN, Germany) (Zhang and Gant, 2005; Kainkaryam et al., 2010; Devi et al., 2016; Zhao et al., 2017). The success of DNA removal was confirmed by PCR of the 16S rRNA gene with primers 27F and 1492R (DeLong, 1992) (Supplementary Table S1). The reaction contained 5 μL of the 10x LA Taq buffer (Mg^+2^), 4 μL of dNTPs (2.5 mM), 1 μL of primer 27F (10 μM), 1 μL of primer 1492R (10 μM), 1 μL of the template DNA/RNA, 0.5 μL of the LA Taq polymerase, and 37.5 μL of ddH_2_O. The PCR conditions used were 94°C for 30 s and 28 cycles of 55°C for 30 s and 72°C for 90 s. Again, the integrity and quantity were assessed using NanoDrop 2000 Spectrophotometer and Agilent Bioanalyzer 2100 (Agilent Technologies, United States). The depletion of ribosomal RNA before cDNA synthesis was performed using the Ribo-Zero kit (Epicentre Biotechnologies, United States) for meta-bacteria. The mRNA was fragmented using the Ambion RNA fragmentation kit (Ambion, United States), and double-stranded cDNA was generated using the Qubit dsDNA HS Assay Kit (Invitrogen, United States). Subsequently, four mRNA libraries were constructed, evaluated using the Agilent Bioanalyzer 2100, and sequenced using 2 × 125 paired-end reads on an Illumina HiSeq2500 sequencer using the HiSeq SBS Kit v4 (Illumina, United States). Approximately 5 Gbp of clean data was targeted for each library. There were 57,246,312; 38,944,960; 43,907,548; and 47,725,396 reads of total clean data obtained, and 57,189,768; 38,862,480; 43,867,792; and 47,697,494 reads of clean data were left after rRNA removal for the mono- and co-culture performed with initial pH of 7.0 and 8.3.

### RNA-Seq Data Analysis

Raw reads were filtered to remove low quality reads using Seqtk^1^ with the following standards: (1) reads with adaptors were removed; (2) reads containing more than 50 bases with low quality (Q20) were removed; (3) reads with more than 3 N bases were removed; (4) low quality bases or N bases assigned at the 3′ tail were removed; and (5) reads shorter than 20 bp were removed. To eliminate all ribosomal RNA sequences, reads mapping to the rRNA (5S rRNA, 16S rRNA, and 23S rRNA) of YYL and MLY1 were removed, and the remaining clean reads were used for the subsequent analysis.

All the clean reads from the mono- and co-culture systems were pooled and assembled using the Trinity assembly algorithm for Primary UniGene and CAP3 EST for Final UniGene (First_contig and Second_contig) (Huang and Madan, 1999; Grabherr et al., 2011). The acquired Final UniGene sequences were searched against the NCBI non-redundant (nr) database (March 2014) using BLASTx (version BLAST-2.2.28+) (Altschul et al., 1990) for the detection of protein-coding genes with the parameter *e*-value < 10^-30^, identity >60%, and genes were identified using self-developed Perl scripts; the maximum overlap against adjacent genes was 100 bp. Subsequently, further redundancy was removed using CD-HIT-EST v4.6 (Li and Godzik, 2006) with a sequence identity threshold of 99% in every 1000 bp (Wong et al., 2015). A collection of 9,483 non-redundant genes were used as a reference genome for the differential gene analysis.

Clean reads from each condition were aligned to the whole-body transcriptome of YYL. The normalized output for each gene expression was calculated as Reads Per Kilobase per Million mapped reads (RPKM) (Mortazavi et al., 2008). For Differentially Expressed Gene (DEG) sets, hierarchical clustering analysis was performed using the complete linkage and Euclidean distance as a measure of similarity. All data analyses and visualization of DEGs were conducted using R3.0.2^2^. Gene expression under different pH conditions in the mono- and co-culture systems were compared, and the differential expression was considered as a fold-change ≥2 and false discovery rate (FDR) < 0.05.

All the gene sequences were searched against the Gene Ontology (GO) (*e*-value < 10^-5^), Kyoto Encyclopedia of Genes and Genomes (KEGG), and evolutionary genealogy of genes Non-supervised Orthologous Groups (eggNOG) using BLASTx (version: BLAST-2.2.28^+^) (Altschul et al., 1990; Ashburner et al., 2000). To confirm which GO terms and metabolic pathways from the mono- and co-culture systems responded to the different pH conditions, GO and KEGG enrichment analyses were performed using DAVID (Huang et al., 2009). The significantly enriched GO terms and KEGG pathways in the DEGs were identified with the hypergeometric test in the entire genome background. The calculated *p*-value was corrected by the Bonferroni method with a threshold of *p*-value ≤ 0.05 to define the GO terms and KEGG pathways as significantly enriched. Subsequently, the major biological functions, the most important biochemical metabolic pathways, and signal transduction pathways with differentially expressed genes (DEGs) were identified by GO and pathway significance enrichment.

Sequencing data are available in the NCBI database under the accession numbers SRR5723771, SRR5723772, SRR5723773, and SRR5723774.

## Results

### THF Degradation in the Incubation Systems

**FIGURE 1:**
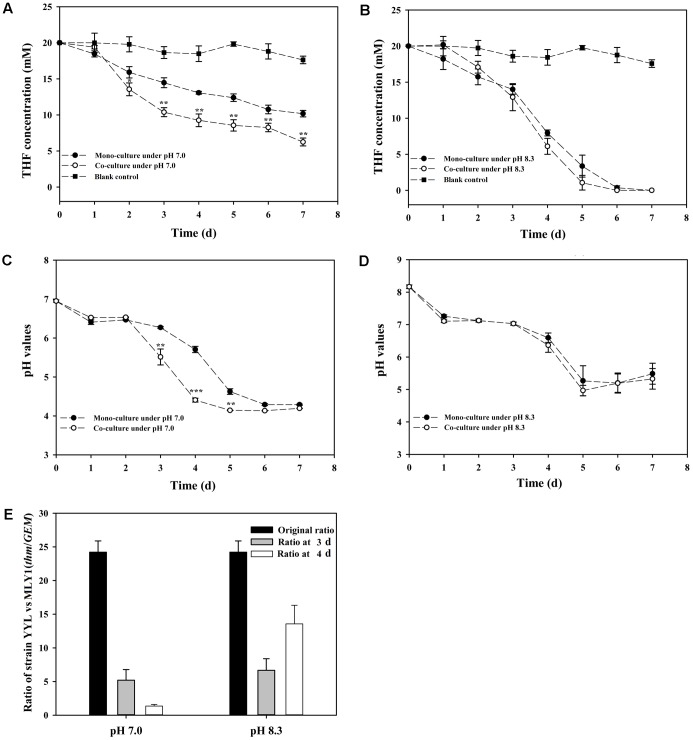
Tetrahydrofuran (THF) degradation curves under the initial pH of 7.0 **(A)** and 8.3 **(B)**, pH change curves under the initial pH of 7.0 **(C)** and 8.3 **(D)**, ratio of strain YYL to strain MLY1 **(E)** in the mono-culture and co-culture systems under pH stress (initial pH = 7.0) and the optimal initial pH of 8.3. Significance was analyzed by the Student’s *t*-test (*n* = 4, ^∗^*P* < 0.05, ^∗∗^*P* < 0.01, ^∗∗∗^*P* < 0.001).

As previously mentioned, the pH is a significant variable affecting the THF degradation efficiency of strain YYL, and its optimal initial pH for THF degradation is 8.3 (Yao et al., 2009). The degradation efficiency of strain YYL at the sub-optimal initial pH of 7.0 can be improved by co-culture of YYL with MLY1. Therefore, strains YYL and MLY1 were mono- or co-cultured at initial pH of 7.0 and 8.3 and subjected to degrading physiological analysis (**Figure 1**). When the THF concentration and pH between the co-culture and mono-culture systems were compared, there was no significant difference found under the initial pH of 8.3, but a lower THF concentration and pH were detected under the initial pH of 7.0 in the co-culture than in the mono-culture (**Figures 1A–D**). Meanwhile, the ratio of strain YYL/strain MLY1 was lower when under the initial pH of 7.0 than that observed under the initial pH of 8.3 (**Figure 1E**), suggesting that strain MLY1 plays a more significant role under low pH conditions. Additionally, a more obvious synergistic promotion of the THF degradation was detected between strain YYL and MLY1 with low pH stress (initial pH = 7.0).

### Transcriptional Response of Strain YYL to pH Stress in Mono-culture

**FIGURE 2:**
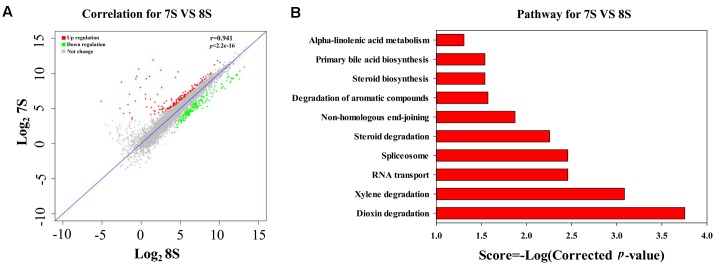
Correlation plot **(A)** and metabolism pathway (KEGG) analyses **(B)** of differentially expressed genes (DEGs) from strain YYL under the initial pH of 7.0 and 8.3. 7S and 8S represent the mono-culture of strain YYL under the initial pH of 7.0 and 8.3, respectively.

As discussed above, the low initial pH of 7.0 was unfavorable for the THF degradation by strain YYL. To explore the effects of pH on the gene expression of strain YYL, transcriptional differences in the mono-culture systems of strain YYL under the initial pH of 7.0 and 8.3 were compared. As shown in **Figure 2A**, clear differences were observed in the general patterns of the transcriptional responses to the two-different pH. Growing strain YYL under the initial pH of 7.0 led to both increases and decreases in gene expression when compared to growing at the initial pH of 8.3. To explore the functions of the DEGs from strain YYL, KEGG, and GO annotation were analyzed.

**FIGURE 3:**
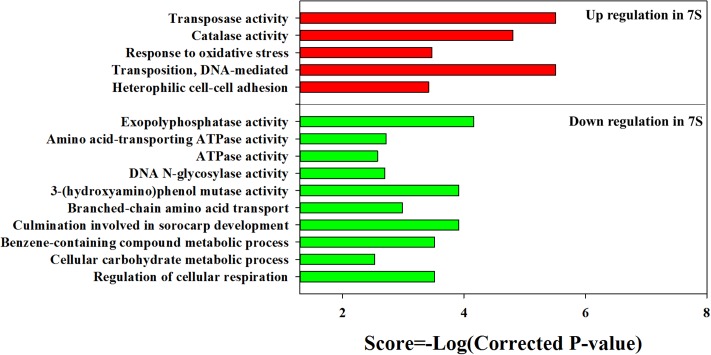
Gene Ontology (GO) enrichment results for DEGs from strain YYL in 7S vs. 8S. 7S and 8S represent mono-culture under the initial pH of 7.0 and 8.3, respectively.

The KEGG enrichment analysis revealed that steroid degradation and biosynthesis, degradation of the aromatic compounds, xylene, and dioxin, RNA transport, spliceosome, non-homologous end-joining, alpha-linoleic acid metabolism, and bile acid biosynthesis were up-regulated when strain YYL was grown under the initial pH of 7.0 (**Figure 2B**). Also, the GO functional analysis showed that pH stress (initial pH = 7.0) induced up-regulation of genes involved in the transposase and catalase activity, response to oxidative stress, transposition, and heterothallic cell-cell adhesion, with down-regulation of the ATPase activity, exopolyphosphatase activity, DNA N-glycosylase activity, branched-chain amino acid transport, and cellular carbohydrate metabolic processes occurring (**Figure 3**).

In summary, strain YYL responded to the low initial pH (7.0) stress by enhancing steroid concentration, anti-oxidation, additional compound degradation, translation, and mutation activity. Meanwhile, the low initial pH (7.0) stress decreased the ATPase activity, carbohydrate metabolic activity, and cell respiration of strain YYL.

### Transcriptional Response of Strain YYL to Co-culture Under Different pH Values

**FIGURE 4:**
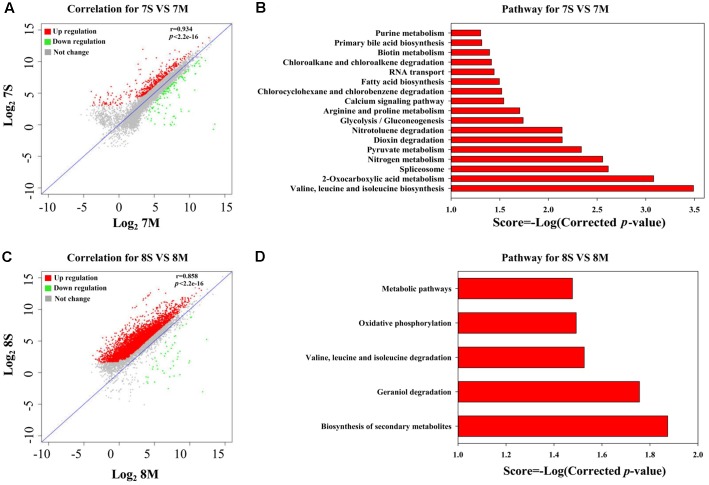
Analysis of DEGs between mono-culture and co-culture in strain YYL. Correlation plot **(A)** and metabolism pathway (KEGG) analyses **(B)** under the initial pH of 7.0. Correlation plot **(C)** and metabolism pathway (KEGG) analyses **(D)** under the initial pH of 8.3. 7S and 7M represent the mono-culture and co-culture under the initial pH of 7.0, respectively. 8S and 8M represent the mono-culture and co-culture under the initial pH of 8.3, respectively.

According to the THF degradation curves (**Figures 1A,B**), strain MLY1 exhibited a synergistic promotion of the THF degradation by strain YYL when grown with initial pH of 7.0 but had no such effect when grown with initial pH of 8.3. Therefore, we hypothesized that strain MLY1 would induce different effects on the gene expression of strain YYL in co-cultured systems grxown at a different pH. As shown in **Figures 4A,C**, the correlation of the gene expression from strain YYL between the mono-culture and co-culture indicates that strain MLY1 influences the gene expression of strain YYL.

The DEGs from strain YYL derived from comparing mono- and co-culture under the initial pH of 7.0 were also analyzed by KEGG and GO annotation. As shown in **Figures 4B,D**, significantly different KEGG pathways were up-regulated in mono-culture when grown with initial pH of 7.0. The depleted KEGG pathways from the co-culture system included RNA transport, primary bile acid biosynthesis, glycolysis, 2-oxocarboxylic acid metabolism, and amino acid biosynthesis. The GO functional analysis revealed that the depleted GO functions from the co-culture system included 113 terms, such as the fatty acid biosynthesis process, response to acid, amino acid biosynthesis process, ATPase activity, and malate dehydrogenase activity (Supplementary Figure S1A); the enriched GO functions from the co-culture system included 25 terms, such as the preribosome, phosphoprotein phosphatase activity, and isopentenyl diphosphate biosynthetic process (Supplementary Figure S1B).

**FIGURE 5:**
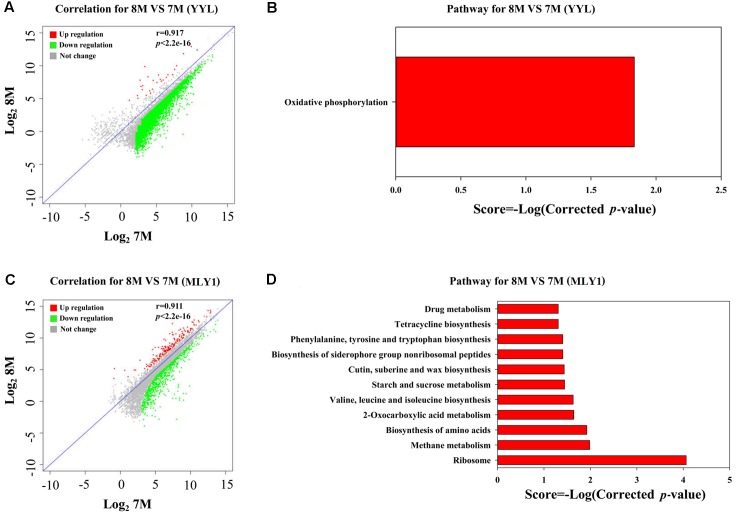
Analysis of DEGs between the initial pH of 7.0 and 8.3 in the co-culture systems. Correlation plot **(A)** and metabolism pathway (KEGG) analyses **(B)** in strain YYL. Correlation plot **(C)** and metabolism pathway (KEGG) analyses **(D)** in strain MLY1. 7M and 8M represent co-culture under the initial pH of 7.0 and 8.3, respectively.

In the co-culture grown with initial pH of 8.3, the depleted KEGG pathways from strain YYL included metabolic pathways, oxidative phosphorylation, valine, leucine, and isoleucine degradation, geraniol degradation, and biosynthesis of secondary metabolites (**Figure 5D**). The GO functional analysis revealed that the depleted GO functions from the co-culture system included nine terms, such as RNA binding, proton-transporting ATP synthase activity, and growth (Supplementary Figure S2); the enriched GO function terms from the co-culture system included only the ribonucleo-protein complex and cytosolic large ribosomal subunit.

In summary, strain MLY1 imposed more effects on the gene expression of strain YYL when grown with initial pH of 7.0 than when grown with initial pH of 8.3. With initial pH of 7.0, strain YYL exhibited a lower level of amino acid biosynthesis, fatty acid biosynthesis, ATPase activity, response to acid, and translation activity because of the influence of strain MLY1; with initial pH of 8.3, strain YYL exhibited a lower level of the functions involved in cellular structure and biological function, such as cell wall synthesis, RNA binding, and cell growth.

### Transcriptional Response to pH Stress in Co-culture

In the mono-culture system, pH stress (initial pH = 7.0) induced significant alterations in the gene expression of strain YYL. To explore the differences in gene expression between the two pH values in the co-culture systems, the transcriptomes of strain YYL and MLY1 were analyzed.

As shown in **Figure 5A**, most of the DEGs from strain YYL were up-regulated when YYL was grown with initial pH of 7.0 in the co-culture system. The KEGG enrichment analysis of DEGs indicated that oxidative phosphorylation was up-regulated in strain YYL because of the pH stress (initial pH = 7.0) (**Figure 5B**). The GO functional analysis showed the enrichment of GO function terms involved in the structural constituent of the ribosome, ATP synthesis, translation, and growth; the depleted GO function terms included rRNA binding, ribosome, ribonucleo-protein complex, and cytosolic large ribosomal subunit (Supplementary Figure S3).

As shown in **Figure 5C**, strain MLY1 in the co-culture system exhibited different gene expression when grown under the initial pH of 7.0 and 8.3. The KEGG analysis of differential genes showed the up-regulation of pathways from strain MLY1 grown with initial pH of 7.0 such as the biosynthesis of amino acids, 2-oxocarboxylic acid metabolism, sucrose metabolism, and ribosome (**Figure 5D**). The GO functional analysis revealed only two GO functional terms that were up-regulated in strain MLY1 grown with initial pH of 7.0 (Supplementary Figure S4).

In summary, the effects of low initial pH (7.0) stress on the gene expression of strain YYL were less notable in the co-culture system than in the mono-culture system. Meanwhile, strain MLY1 enhanced 2-oxocarboxylic acid metabolism and the synthesis of amino acids, ATP, and ribosomes to help strain YYL respond to the low initial pH (7.0) stress in the co-culture system.

## Discussion

The response to pH stress plays a vital role in the survival of Gram-positive bacteria, and the mechanisms utilized by the gram-positive bacteria in response to pH stress operate in different ways (Cotter and Hill, 2003). However, the THF-degrading strain YYL exhibits weak resistance to low pH stress, with a low growth rate and THF degradation efficiency under low pH stress (Yao et al., 2009). In this study, two genes involved in anti-oxidation and mutation in strain YYL were unregulated when YYL was grown with initial pH of 7.0 suggesting that this pH renders an environmental stress on strain YYL. To deal with this stress, the synergistic relationships among microorganisms can be beneficial in the natural environment (Ma et al., 2016; Ren et al., 2016). Based on our analysis of the THF degradation efficiency, a synergistic relationship between strain YYL and the non-THF-degrading strain MLY1 exists when strain YYL experiences pH stress (initial pH = 7.0) but not when strain YYL is not stressed (initial pH = 8.3).

**FIGURE 6:**
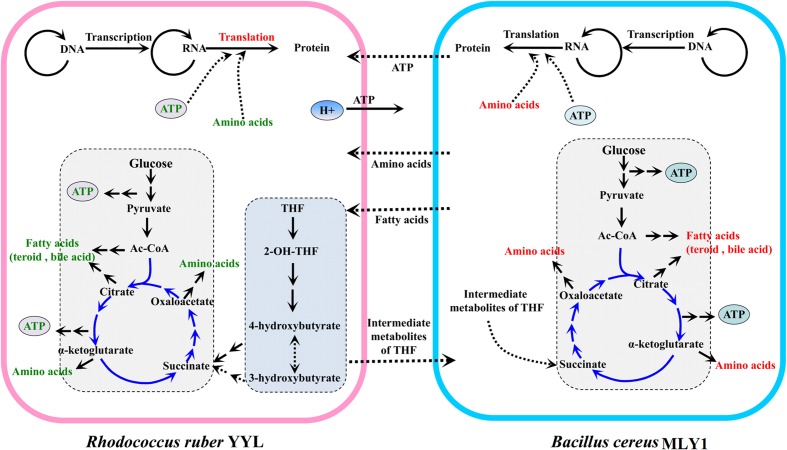
The schematic mechanism of the interspecific interactions between strain YYL and strain MLY1 under pH stress (initial pH = 7.0) during THF biodegradation. Strain YYL degrades THF to intermediates, providing carbon sources for strain MLY1. THF degradation is an acid-producing process, and the protons produced need be transported to the extracellular space, a process dependent upon ATP. Low pH stress causes a decrease in the metabolism of ATP, fatty acids, and amino acids in strain YYL, while strain MLY1 provides the ATP, fatty acids, and amino acids for strain YYL to relieve the pH stress. Red text represents up-regulated metabolism; green text represents down-regulated metabolism. The middle-dashed arrows point in the direction of metabolic flow between strain YYL and MLY1.

Based on these results, we propose a model for the cooperation between strains YYL and MLY1, shown in **Figure 6**. In co-culture system with THF as the sole carbon source, THF is degraded by YYL, and the easily usable intermediates are utilized by MLY1 as carbon sources (**Figure 6**). The unfavorable pH environment causes a significant change in the expression of genes involved in the metabolism of fatty acids required for lipid synthesis, ATP for energy, and amino acids for translation in strain YYL (**Figure 3** and Supplementary Figure S1A), which is lethal for YYL if the pH stress cannot be relieved. THF degradation is an acid-producing process, and the protons produced need be transported out of the cell, a process that is dependent upon ATP (Buch-Pedersen et al., 2009) (**Figure 6**). Fortunately, the symbiotic strain MLY1, surviving on the intermediates of the THF degradation, provides lipids, ATP, and amino acids for strain YYL to relieve the pH stress (**Figure 6**). Based on the above interactions between strain YYL and MLY1, the symbiotic system is stable to completely degrade the THF.

### Strain MLY1 Could Contribute Fatty Acids and Lipids to Strain YYL

In response to pH stress (initial pH = 7.0), strain YYL up-regulates the genes involved in steroid biosynthesis and degradation and bile acid biosynthesis. Steroids and bile acids are both sterols, and interactive transformation exists between the steroids and bile acids in the genus *Rhodococcus* (Donova, 2007; Yam et al., 2011). Up-regulation of steroid metabolism plays a significant role in the response to environmental stress by *Rhodococcus* sp. (Larkin et al., 2006; Orro et al., 2015). This may be because of the fact that steroid metabolism is related to energy supply and membrane structure in microbial cells (Haussmann et al., 2013). In addition, previous literature has reported that most of the enzymes in charge of steroid transformation belong to the family of cytochrome P450 monooxygenases (P450s) in the Actinobacteria (Shtratnikova et al., 2016); this enzyme activity can be inhibited by THF (Urlacher and Girhard, 2012). Therefore, the THF inhibition of the P450s in strain YYL grown with initial pH of 7.0 might induce the up-regulation of the genes encoding for the P450s to ensure adequate P450 function for steroid transformation, for instance.

At initial pH of 7.0, the co-culture of strain YYL and strain MLY1 causes down-regulation of the bile acid and fatty acid biosynthesis necessary for lipid production in strain YYL (Supplementary Figure S1A). Simultaneously, strain MLY1 up-regulates the genes involved in the biosynthesis of fatty acids and lipids, including cutin, suberine, and wax (**Figure 5**). We propose that strain MLY1 might contribute the fatty acids and lipids to help strain YYL respond to the low pH stress in the co-culture system.

### Flow of ATP from Strain MLY1 to Strain YYL

Bacteria frequently encounter environmental stress that generates a severe demand for ATP (Kobayashi et al., 1986); for example, exposure to low pH in the case of *Escherichia coli* requires ATP (Bearson et al., 1997). However, strain YYL exposure to pH stress (initial pH = 7.0) causes down-regulation of the genes involved in ATP production, such as the ATPase, cell respiration, and carbohydrate metabolic functions. Generally, ATPase activity, a good marker for the condition of the cell membrane and its enzymatic activity (Garbay and Lonvaudfunel, 1994), is essential for growth; the ATPase counteracts variations in the cytoplasmic pH by pumping protons out of the cells during low pH stress (Kullen and Klaenhammer, 1999; Kuhnert et al., 2004). The down-regulation of ATPases by strain YYLunder low pH stress (initial pH = 7.0) is not beneficial for responding to the pH stress. Therefore, the down-regulation of genes involved in ATP production likely explains the poor efficiency of THF degradation by strain YYL when grown at initial pH of 7.0.

The lack of ATPase activity and extrusion of protons during low pH stress in strain YYL leads YYL to require adequate support from strain MLY1. Comparing strain YYL co-cultured with strain MLY1 using the optimal pH (initial pH = 8.3), we noted the down-regulation of genes involved in ATP synthesis coupled with proton transport and the tricarboxylic acid cycle in the strain YYL co-cultured under pH stress (initial pH = 7.0) (**Figure 3**). Simultaneously, strain YYL up-regulated the genes involved in starch and sucrose metabolism, which could generate ATP in response to the low pH stress.

### Strain MLY1 Could Provide Amino Acids and Synthetic Proteins to Strain YYL

During environmental stress, organisms must tightly regulate the activity of translation because of the high-energy consumption of protein synthesis (Sorensen and Loeschcke, 2007; Yamasaki and Anderson, 2008). To deal with pH stress (initial pH = 7.0), strain YYL up-regulates the genes responsible for RNA transport but down-regulates the genes responsible for amino acid transport. These alterations in gene expression in strain YYL might illustrate a significant reprogramming of protein translation for strain YYL to respond to the pH stress.

In a synergistic relationship of bacteria, the exchange of amino acids frequently occurs (Nobu et al., 2015). When under pH stress (initial pH = 7.0), co-culture with strain MLY1 causes the down-regulation of valine, leucine, and isoleucine biosynthesis in strain YYL (Supplementary Figure S1B). Simultaneously, strain MLY1 up-regulates the genes involved in the biosynthesis of amino acids and translation (**Figure 5D** and Supplementary Figure S4). It is likely that strain MLY1 provides amino acids and some synthetic proteins for strain YYL in the co-culture system.

In summary, this work demonstrates that pH stress imposes an inhibitory effect on the THF-degrading strain YYL. Transcriptome analysis reveals that strain YYL up-regulates the genes involved in anti-oxidation, mutation, steroid and bile acid metabolism, and translation while simultaneously down-regulating the genes involved in ATP production when experiencing the low pH stress. MLY1 has no THF degradation activity, but it could provide fatty acids, ATP, and amino acids for strain YYL in response to the pH stress in the co-culture systems which might relieve the inhibition of strain YYL.

## Limitation Statement

The results reported in this article are only preliminary as four biological replicates were pooled for each condition investigated prior to sequencing.

## Author Contributions

Performed experiments: ZuL and ZH. Analyzed data: ZuL, ZH, HH, and XR. Conceived and designed experiments: ZuL, ZH, HH, XR, AO, and ZhL. All authors have agreed to be accountable for all aspects of the work in ensuring that questions related to the accuracy or integrity of any part of the work are appropriately investigated and resolved.

## Conflict of Interest Statement

The authors declare that the research was conducted in the absence of any commercial or financial relationships that could be construed as a potential conflict of interest.

## Footnotes

1 http://github.com/lh3/seqtk

2 http://www.r-project.org
