# Supplementary material for: pH Stress-Induced Cooperation between Rhodococcus ruber YYL and Bacillus cereus MLY1 in Biodegradation of Tetrahydrofuran
Source: Front Microbiol. 2017 Nov 21;8:2297. doi: 10.3389/fmicb.2017.02297 (PMC5702389; doi:10.3389/fmicb.2017.02297)
Supplement: Supplementary file 1 [file Data_Sheet_1.pdf]

Supplementary Material

pH Stress-induced Cooperation between *Rhodococcus ruber* YYL and *Bacillus cereus* MLY1 in Biodegradation of Tetrahydrofuran

Zubi Liu[1+], Zhixing He[2+], Hui Huang[1], Xuebin Ran[1], AdebanjoOmosalewa Oluwafunmilayo[1], Zhenmei Lu[1*]

[1]College of Life Sciences, Zhejiang University, Hangzhou, China;
[2]College of Basic Medical Science, Zhejiang Chinese Medical University, Hangzhou, China

"+" Contributed equally to this work

"*"Corresponding author: Zhenmei Lu, Ph.D., Institute of Microbiology, College of Life Sciences, Zhejiang University, Hangzhou, Zhejiang, 310058, China

Phone: +86-0571-88206279, Fax: +86-0571-88206485, Email: lzhenmei@zju.edu.cn

Supplementary Table

SupplementaryTable 1. Primers used in this study.

Name	Sequence (5’-3’)
thm FP	CGAGTTCATCCAAGTAGT
thm RP	GAAGTCTCTATCCACTGAT
GerM FP	ATTGATGAATCGGACCTT
GerM RP	GATGAACAAGTAGCAGATG
27F	AGAGTTTGATCCTGGCTCAG
1492R	GGTTACCTTGTTACGACTT

Supplementary Figures

(A)

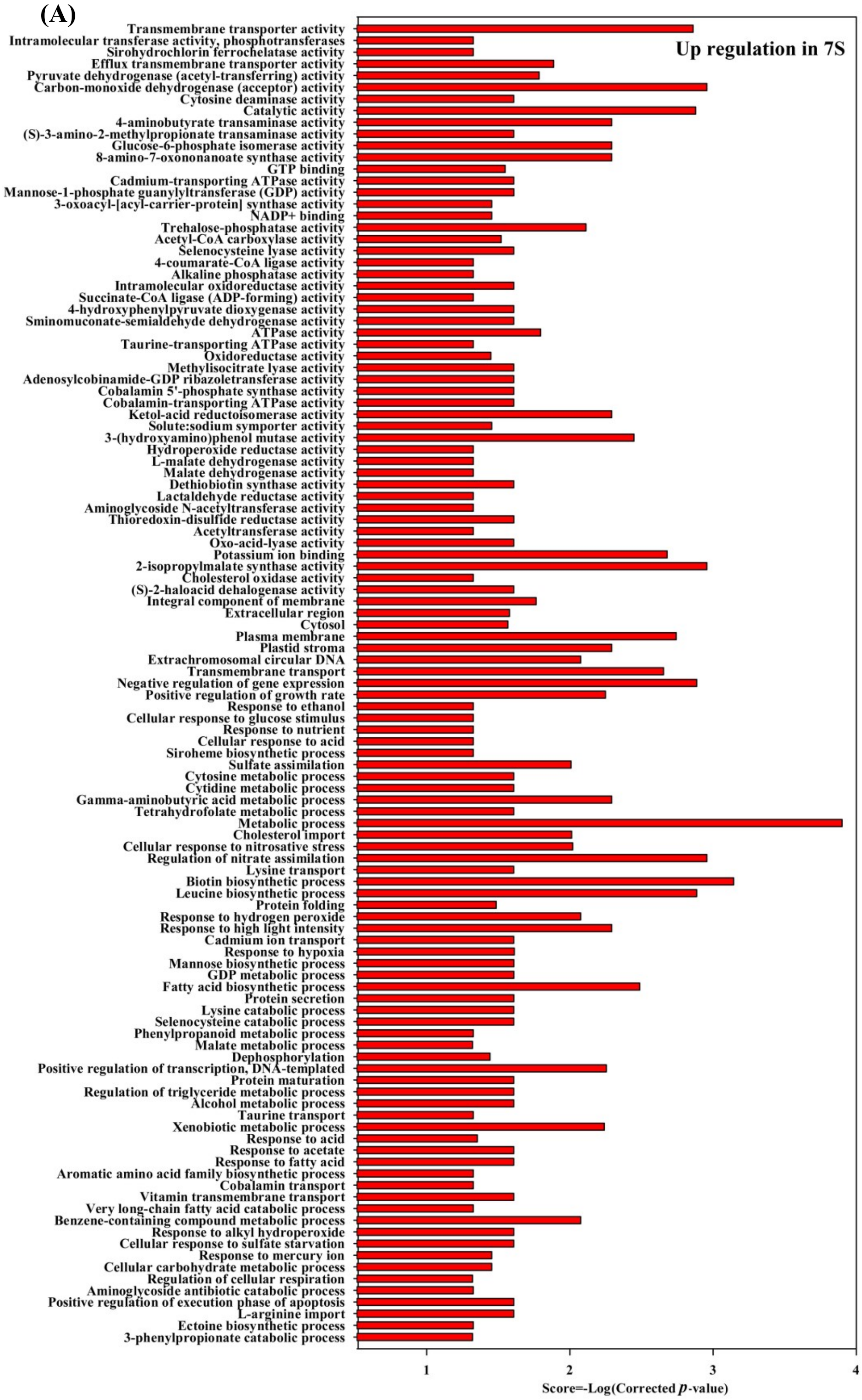

(B)

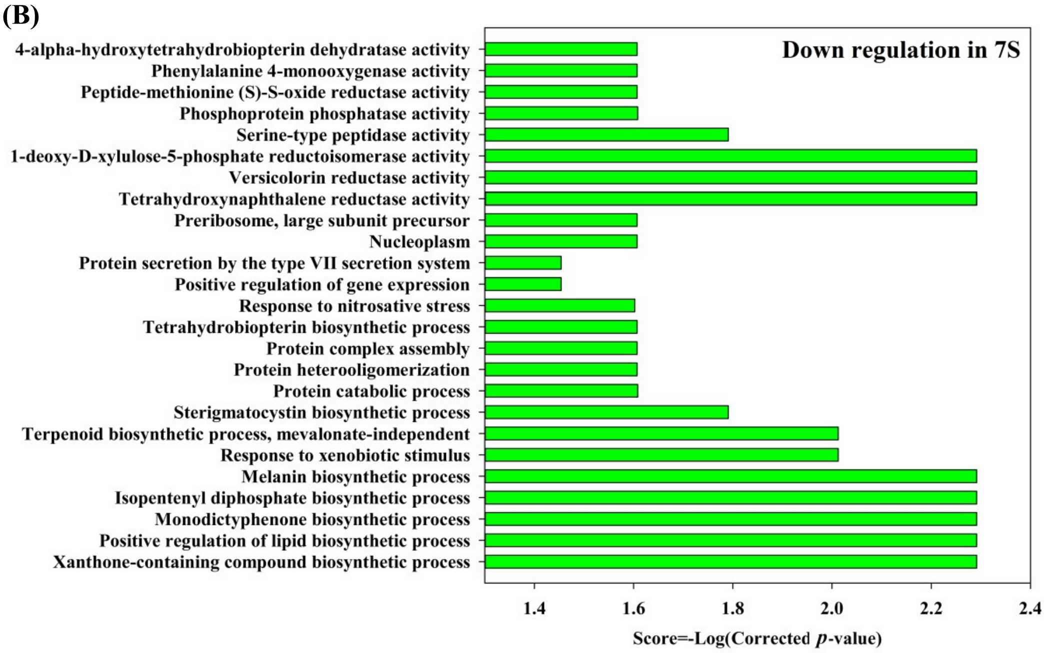

Supplementary Figure1. GO enrichment result of different expression genes of strain YYL in 7S *vs* 7M. 7S and 7M represent mono-culture and co-culture under initial pH 7.0, respectively. (A) Up-regulation in 7S; (B) Down-regulation in 7S.

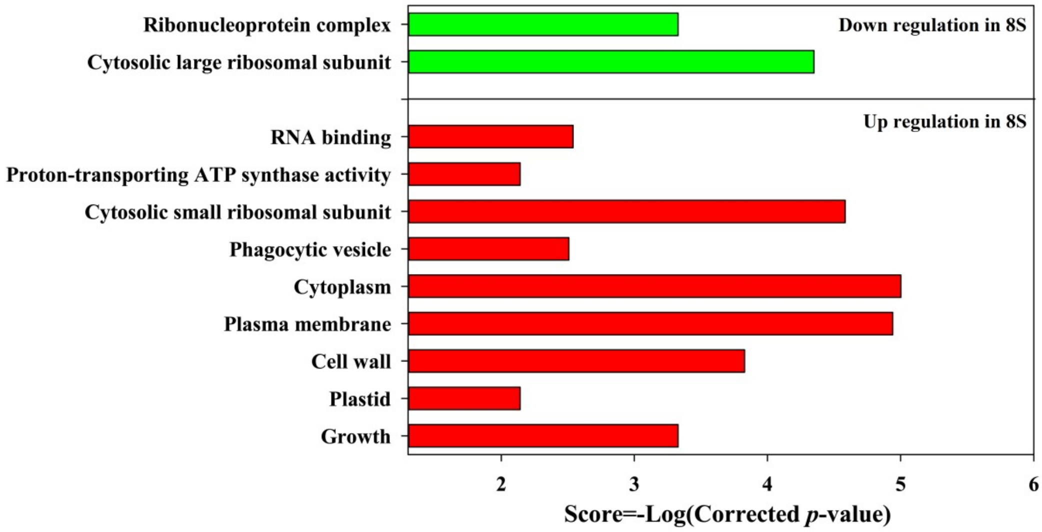

Supplementary Figure2. GO enrichment result of different expression genes of strain YYL in 8S *vs* 8M. 8S and 8M represent mono-culture and co-culture under initial pH 8.3, respectively.

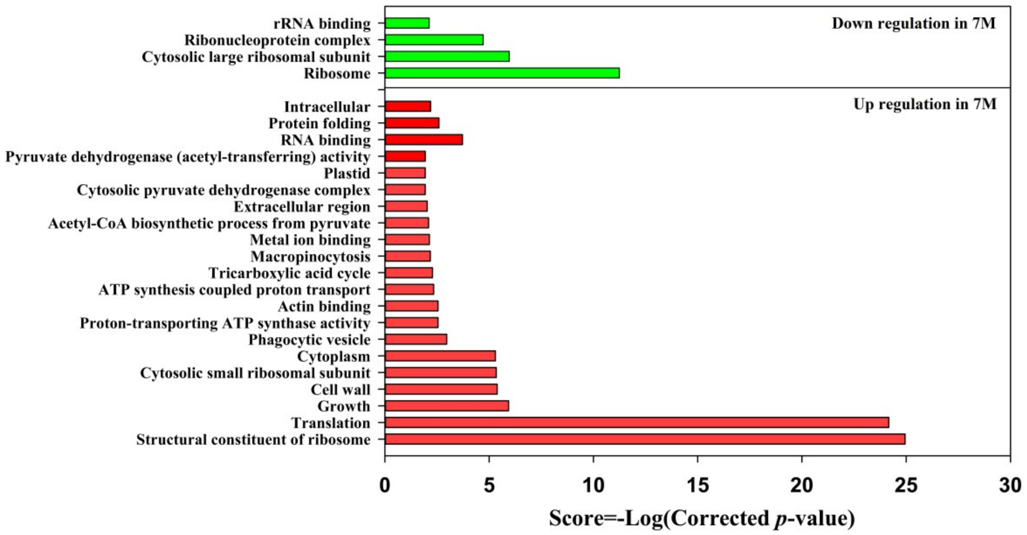

Supplementary Figure3. GO enrichment result of different expression genes of strain YYL in 7M *vs* 8M. “7M”, “8M” represents co-culture under initial pH 7.0 and pH 8.3, respectively.

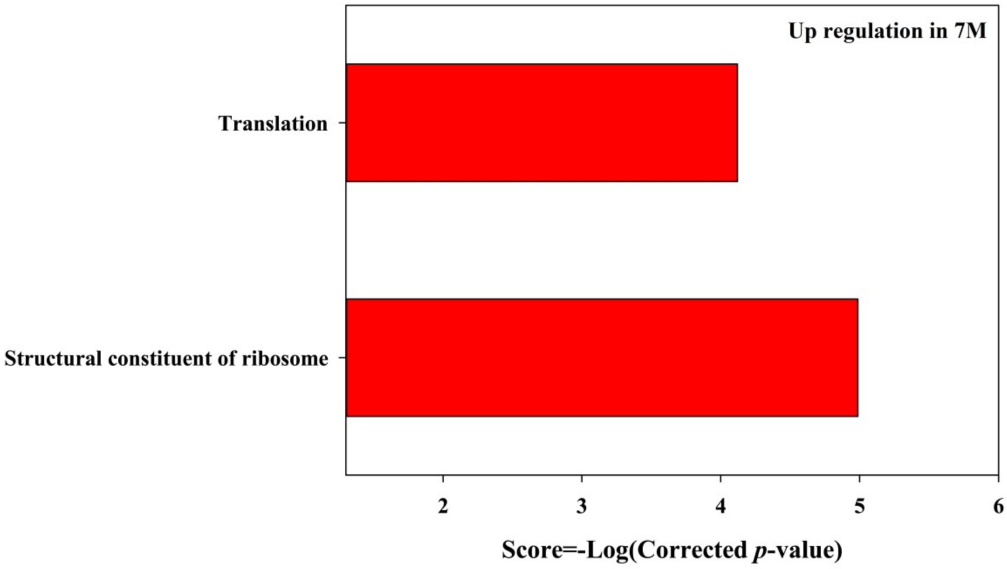

Supplementary Figure4. GO enrichment result of different expression genes of strain MLY1 in 7M *vs* 8M. 7M and 8M represent co-culture under initial pH 7.0 and pH 8.3, respectively.
